# Intestinal Obstruction with Abnormal Development of Colon

**Published:** 1916-03

**Authors:** 


**Intestinal Obstruction With Abnormal Development of Colon.** W. J. Barclay, New Zealand Med. Jour., Dec., 1915, reports a case in a man aged 22, 3 days constipation, pulse 84, temperature 98, swelling in region of caecum, alternately hardening and relaxing. Vomiting. The caecum was exposed, evacuated through an incision and closed, with double row of sutures. From the still distended caecum, the colon passed at once to the left, behind the small intestines, being obstructed by fibrous tissue continuous with the root of the mesentery, 4 inches above the caecum. Some bands were dissected off but it was impossible to free the colon so that the contents could be easily pressed past the obstruction. The healthy appendix was therefore stitched to the abdominal wall to afford a fistula. Death followed in 41 hours from peritonitis, though there was temporary relief. Post mortem, it was found that the caecum and small intestine had a common mesentery, the great omentum hanging free from the stomach and without attachment to the colon at any place.

